# RELATIONSHIPS BETWEEN PHYSICAL/MENTAL MULTIMORBIDITY AND DEPRESSIVE SYMPTOMS AMONG OLDER BLACK AMERICANS

**DOI:** 10.1093/geroni/igac059.2648

**Published:** 2022-12-20

**Authors:** Seungjong Cho, Tyrone Hamler

**Affiliations:** Texas Tech University, Lubbock, Texas, United States; Case Western Reserve University, Cleveland, Ohio, United States

## Abstract

The purpose of this study was to identify the association between physical/mental health problems and depressive symptoms among older Black Americans (age ≥ 65) using the 2018 Health and Retirement Study (N = 812). People with multiple medical conditions face substantial emotional difficulties including depressive symptomatology. The study of depressive symptoms among older Black Americans is an emerging area of research. Several existing studies reported a higher prevalence of depressive symptoms among this population. They also experience a disproportionate burden of multimorbidity, both physically and mentally. For statistical analysis, depressive symptoms were measured with the eight-item CES-D scale (M = 1.514, SD = 1.916). Physical multimorbidity was measured by asking whether respondents had ever been diagnosed with seven diseases (high blood pressure, diabetes, cancer, lung disease, heart disease, stroke, and arthritis; M = 2.876, SD = 1.284). Mental multimorbidity was measured by asking whether they had ever been diagnosed with three diseases (clinical depression, Alzheimer’s Disease, or other memory impairment including dementia; M = .228, SD = .467). Covariates included gender, marital status, and income (logged). A negative binomial regression showed that (a) higher numbers of physical health problems were associated with higher depressive symptoms (Incidence rate ratios [IRR] = 1.073, p = .048); (b) higher number of mental health problems were also related to higher depressive symptoms among older Black Americans (IRR = 2.077, p < .001). These findings underscore the importance of identifying and managing depressive symptoms among older Black Americans, who present with greater physical/mental health multimorbidity.

